# Relevance of the 21-gene expression assay in male breast cancer: A systematic review and meta-analysis

**DOI:** 10.1016/j.breast.2022.04.009

**Published:** 2022-04-28

**Authors:** Matthew G. Davey, Ciara M. Davey, Luis Bouz, Eoin Kerin, Carson McFeetors, Aoife J. Lowery, Michael J. Kerin

**Affiliations:** Department of Surgery, The Lambe Institute for Translational Research, National University of Ireland, Galway, H91YR71, Ireland

**Keywords:** Male breast cancer, Cancer genomics, Precision oncology, Personalised medicine

## Abstract

**Introduction:**

The 21-gene assay provides prognostication for estrogen receptor positive, human epidermal growth factor receptor-2 negative (ER+/HER2-) early female breast cancer patients. This signature has not been validated in male breast cancer (MBC).

**Methods:**

A systematic review and meta-analysis was performed in accordance to the PRISMA guidelines. Retrospective cohort studies comparing 21-gene assay scores in female and MBC were included. Dichotomous variables were pooled as odds ratios (OR) and associated 95% confidence intervals (CI) using the Mantel–Haenszel method.

**Results:**

Six studies including 176,338 patients were included (mean age of 63.4 years, range: 33–88). Of these, 1.0% had MBC (1826/176,338) and 99.0% were female patients (174,512/176,338). MBC patients were more likely to have increased tumour stage, nodal involvement, and grade 3 disease (all *P* < 0.001) In MBC patients, the mean score was 18.8 (range: 11–26) vs. 13.4 (range 0–33) in female patients (*P* < 0.001). In MBC patients, 22.4% had scores >30 (408/1822) versus 18.3% in female patients (31,852/174,500). In female patients, 52.0% had scores <18 (90,787/174,500) versus 47.8% in MBC (471/1822). Overall, patients with female patients were as likely to have scores <18 (OR: 1.04, 95% CI: 0.94–1.16), scores 18–30 (OR: 1.12, 95% CI: 1.00–1.26) and scores >30 (OR: 0.69, 95% CI: 0.45–1.07) as MBC patients.

**Conclusion:**

There are similar anticipated scores for female and MBC undergoing 21-gene expression assay testing for early stage, ER+/HER2-breast cancer. In the absence of stage matching, cautious interpretation of these results is required. Validation of the 21-gene assay in MBC is still required.

## Highlights

• Overall, Recurrence Scores (RS) were similar in male and female patients.
• RS testing in male breast cancer (MBC) favoured RS > 30 (P < 0.001).
• Female patients were more likely to have RS < 18 (P < 0.001).
• At meta-analysis, there was no difference in RS among female and MBC patients.
• In the absence of matching, cautious interpretation of RS in MBC is required.

## 1 Introduction

Breast cancer is the commonest cancer diagnosed in women, with an incidence of 12.8% in the western world [1]. Less than 1.0% of breast cancers occur in male patients, which is perceived to be due to the smaller volume of breast parenchyma in males, as well as less endogenous estrogen production in males [2]. While clinicopathological (increased age - the mean age at diagnosis is 71 years in cases of MBC), lifestyle (i.e. high body mass indices, and obesity), and genetic parameters (i.e.: BRCA1/2 mutation status), are well reported risk factors for male breast cancer (MBC) [2,3], there have been limited studies evaluating the molecular biology and natural history of MBC. At diagnosis, MBC tnd to be advanced, steroid hormone receptor positive cancers, with invasive ductal carcinoma (IDC) histology, which ultimately require resection with mastectomy and treatment with adjuvant chemoendocrine therapy [4]. Overall, age- and stage-matched MBC patients have poorer clinical and oncological outcomes than female patients [[5], [6], [7]].

The molecular era has transformed our appreciation for the intrinsic biology of breast cancers, facilitating the novel taxonomy of breast cancer into four clinically distinct molecular subtypes [8]. These molecular subtypes may be determined directly using the PAM-50 multigene signature (NanoString Technologies, Seattle, Washington, USA) or using the immunohistochemistry-stained surrogate profiles for estrogen (ER), progesterone (PgR) and human epidermal growth factor receptor-2 (HER2/neu) receptors [8]. Moreover, the 21-gene expression assay (Genomic Health Inc., Redwood City, CA, USA) is routinely used in ER-positive/HER2-negative, lymph node (LN)-negative female breast cancer patients to determine those who will derive the most benefit from systemic chemotherapy prescription [[9], [10], [11]]. Results of reverse-transcriptase polymerase chain reaction (RT-PCR) of 21 selected genes from the paraffin-embedded tumour blocks of female patients diagnosed with ER-positive, HER2-negative, LN-negative breast cancers from the National Surgical Adjuvant Breast and Bowel Project (NSABP) B-20 trial were used to derive the algorithm which is used routinely within the multigene assay, which was then independently validated using data from the NSABP B-14 trial [10,12]. Of note, there were no tumour samples from MBC patients used in the development or validation of the 21-gene signature [9,10] and there are currently no prospective studies looking to evaluate the clinical utility of the 21-gene expression assay in MBC patients. Therefore, the validity of using the 21-gene expression assay within the setting of MBC may be brought into question. Accordingly, the aim of the current study was to perform a systematic review and meta-analysis to compare 21-gene expression assay scores in MBC and female breast cancer patients.

## 2 Methods

We performed a systematic review and meta-analysis in accordance to the Preferred Reporting Items for Systematic Reviews and Meta-Analyses (PRISMA) and MOOSE guidelines [13,14]. Local institutional ethical approval was not required. All authors contributed to formulating the study protocol and it was then registered with the International Prospective Register of Systematic Reviews (PROSPERO): CRD 42021283956.

### 2.1 Population, intervention, comparison, outcomes (PICO)

Using the PICO framework [15], the aspects the authors wished to address using meta-analysis methodology were:

Population –Patients with newly diagnosed ER-positive breast cancer aged 18 years or older without distant metastatic disease who underwent 21-gene expression assay testing (Genomic Health Inc., Redwood City, CA) performed on their resected breast cancer specimen.

Intervention – Any male patient included in this population.

Comparison – Any female patient included in this population.

Outcomes – Primary outcomes included: 21-gene expression assay results conducted on resected breast cancer tissue, including risk group.

### 2.2 Search strategy

A formal electronic search of the *PubMed, EMBASE and Scopus* databases was performed for relevant studies. This search was performed by two independent reviewers (CMD & MGD), using a predetermined search strategy that was designed by the senior authors (AJL & MJK). This search included the search terms: (Oncotype) and (male breast cancer), linked using the Boolean operator ‘AND’. Included studies were limited to the English language and were not restricted by year of publication. Manual removal of duplicate studies was performed, before all titles were screened. Thereafter, studies considered to be appropriate had their abstracts and/or full text reviewed. Retrieved studies were reviewed to ensure inclusion criteria were met for one primary and secondary outcome at a minimum. In cases of discrepancies of opinion a third author was asked to arbitrate (EK). The final search was performed on the June 9, 2021.

### 2.3 Inclusion and exclusion criteria

Studies included were clinical studies comparing patients diagnosed with MBC with or without female breast cancer patients who had undergone 21-gene expression assay testing on their resected cancer specimen. For inclusion in the systematic review and pooled analysis, studies with male patients (with or without female patients) were considered for inclusion. These were then pooled with the MBC RS data from other studies before being analysed and compared to the pooled data from female patients from the other included studies. Studies were required to compare RS testing in both male and female patients before being considered for inclusion in the meta-analysis.

All studies included male patients aged 18 years or greater diagnosed with ER-positive (defined in accordance to the American Society of Clinical Oncology/College of American Pathologists as >1% ER expression on immunohistochemical analysis) and HER2-negative (defined as a score of 0 or 1+ on immunohistochemical staining or HER2-negative following fluorescence in-situ hybridisation) breast cancer on resected histopathological specimen [16,17]. Outcomes of interest included 21-gene expression assay testing and clinicopathological data. Studies including data from patients with advanced breast cancer were excluded. Published abstracts from conference proceedings were excluded, as were case reports, case series reporting outcomes in five patients or less, and editorial articles. In cases where study data overlapped from the same resource (e.g.: the National Cancer Database (NCDB) or Surveillance, Epidemiology, and End Results (SEER) program database), studies were selected at the time of full-text review from inclusion based on the number of male patients reported, with those with a larger number favoured for inclusion.

### 2.4 Data extraction and quality assessment

The following data was extracted and collated from retrieved studies meeting inclusion criteria [1]: First author name [2], year of publication [3], study design [4], country of origin [5], number of patients [6], number of patients with MBC [7], number of patients with female breast cancer [8] median age (and range) at diagnosis [9], mean 21-gene assay [10], 21-gene assay categorization, and [11] clinicopathological data. Risk of bias and methodology quality assessment was performed in accordance to the Newcastle-Ottawa Scale [18].

### 2.5 Statistical analysis

Descriptive statistics were used to determine associations between MBC and female breast cancers and 21-gene assay categories. Data was expressed as dichotomous or binary outcomes, reported as odds ratios (ORs) were expressed with 95% confidence intervals (CIs) following estimation using the Mantel-Haenszel method. Either fixed or random effects models were applied on the basis of whether significant heterogeneity (*I*^*2*^ >50%) existed between studies included in the analysis. Symmetry funnel plots were used to assess publication bias. Statistical heterogeneity was determined using *I*^*2*^ statistics. All tests of significance were two-tailed with *P* < 0.050 indicating statistical significance. Descriptive statistics were performed using the Statistical Package for Social Sciences (SPSS) version 26 (International Business Machines Corporation, Armonk, New York). Meta-analysis was performed using Review Manager (RevMan), Version 5.4 (Nordic Cochrane Centre, Copenhagen, Denmark). MGD performed each statistical analyses.

## 3 Results

### 3.1 Literature search

**Fig. 1:**
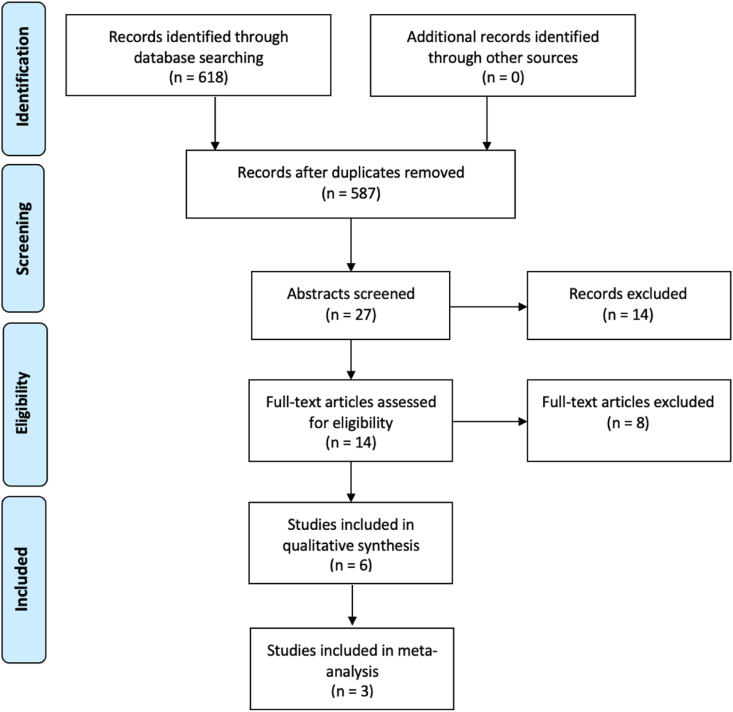
PRISMA flow diagram detailing the systematic search process.

Our initial electronic literature search retrieved 628 studies, of which, 41 duplicate studies were manually removed. The remaining 587 titles were screened for relevance, before 27 studies had their abstracts reviewed and subsequently 14 full-text manuscripts were reviewed in full. In total, 6 studies fulfilled our inclusion criteria and were included in this systematic review (Table 1 & Fig. 1) [[19], [20], [21], [22], [23], [24]]. Of these, 3 studies including 175,903 patients (174,500 female and 1403 males) were included in meta-analyses [21,23,24].

**Table 1:** Details of 6 included studies in this analysis.

Author	Year	Study Type	Country	N	Mean Age (Range)	Male (N)	Female (N)	Scores <18	Scores 18-30	Scores >30	NOS	MA
Turashvili	2018	RC	US	38	70.0 (40–84)	38	0	26	9	3	7	No
Grenader	2014	RC	Israel	2510	65.1 (33–88)	65	2445	1291	960	259	6	Yes
Liu	2020	RC	US	17	N/R	5	12	–	–	–	6	No
Bayani	2021	RC	Multiple	380	N/R	380	0	129	146	106	7	No
Altman	2018	RC	US	46,407	58.1	343	46,064	26,902	16,235	3270	8	Yes
Williams	2020	RC	US	126,986	N/R	995	125,991	63,310	35,054	28,622	7	Yes
–	–	–	–	176,338	63.4 (33–88)	1826	174,512	91,658	52,404	32,260	7^a^	–

N; Number, NOS; Newcastle-Ottawa Scale, MA; Included in meta-analysis, RC; Retrospective cohort, US; United States, N/R; Not Reported.

a Represents median value.

#### 3.1.1 Study characteristics

Six retrospective cohort studies were included in this analysis. Overall, 176,338 patients were included with a mean age of 63.4 years (range: 33–88 years). Of these, 1.0% were MBC patients (1826/176,338) while the vast majority were female patients (99.0%, 174,512/176,338) (5 studies). Of note, MBC patients were more likely to have increased tumour stage, axillary lymph node involvement, and grade 3 disease (all P < 0.001, χ^2^). Table 2 illustrates clinicopathological data for included MBC and females breast cancer patients.

**Table 2:** Clinicopathological and 21-gene expression assay characteristics of the included patients in this study.

Parameter	MBC (N = 1826)	FBC (N = 174,512)	P-value
T1/2	1763 (96.5%)	170,763 (97.9%)	<0.001^d^^,^^a^
T3/4	53 (2.9%)	997 (0.6%)	
Missing	10 (0.6%)	2752 (1.5%)	
Grade 1/2	558 (30.6%)	37,684 (21.6%)	<0.001^d^^,^^a^
Grade 3	242 (13.3%)	7280 (4.2%)	
Missing	1026 (56.1%)	129,548 (74.2%)	
LN negative	1525 (83.7%)	163,089 (93.5%)	<0.001^d^^,^^a^
LN positive	283 (15.5%)	7173 (4.1%)	
Missing	18 (1.0%)	4250 (2.4%)	
Score <18	871 (47.7%)	90,787 (52.0%)	<0.001^d^^,^^b^
Score 18-30	543 (29.7%)	51,861 (29.7%)	
Score >30	412 (22.6%)	31,864 (18.3%)	
21-gene assay score (mean, range)	18.8 (11–26)	13.4 (0–33)	<0.001^d^^,^^c^

MBC; male breast cancer, FBC; female breast cancer, T; tumour stage, LN; lymph node, ER; estrogen receptor, PgR; progesterone receptor, HER2; human epidermal growth factor receptor-2.

a denotes Fisher's Exact test (note: analysis performed on the available data).

b denotes Chi-Square test.

c denotes Independent T-test.

d denotes statistical significance.

#### 3.1.2 21-Gene expression assay

The mean 21-gene expression assay score was 15.2 (range: 0–33). All studies used the traditional numerical categorization as validated by Paik et al. [10]; this considered scores <18 as low-risk, scores of 18–30 as intermediate-risk, and scores >30 as high-risk. Overall, 52.0% had RS < 18 (91,658/176,338), 29.7% had RS 18–30 (52,404/176,338), and 18.5% had RS > 30 (32,660/176,338) (5 studies). Clinicopathological parameters and the 21-gene expression assay in female and MBC patients are illustrated in Table 2.

#### 3.1.3 Male breast cancer and the 21-gene expression assay

In MBC patients, the mean 21-gene expression assay score was only provided by Liu et al. [22]. The mean 21-gene expression assay score for MBC patients was 18.8 (range: 11–26) compared to 13.4 (range: 0–33) in female breast cancer patients (*P* < 0.001).

Overall, there was increased high-risk 21-gene expression assay groups in patients with MBC (P < 0.001, χ^2^): In MBC patients, 22.4% had scores >30 (408/1826) versus 18.3% in female patients (31,852/174,500) (5 studies). Moreover, in female patients, 52.0% had scores <18 (90,787/174,500) versus 47.8% in MBC (871/1826) (5 studies).

**Fig. 2:**
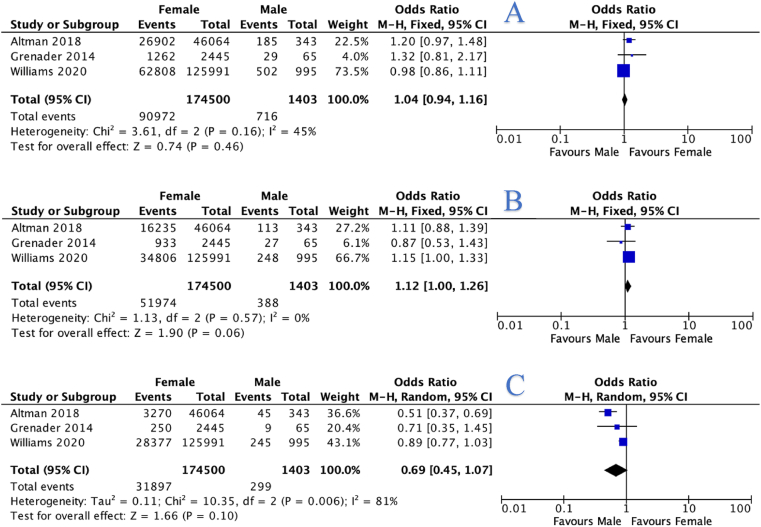
Forest plots illustrating male breast cancer patients were more equally as likely to have a 21-gene expression assay score less than 18 (A), a score of 18–30 (B), and a score >30 (C).

Overall, three studies provided data which was included in meta-analysis [21,23,24]. At meta-analysis, there was a non-significant difference in 21-gene expression assay scores in female and MBC patents: Female patients were as likely to have 21-gene expression assay scores <18 (OR: 1.04, 95% CI: 0.94–1.16, *P* = 0.460, *I*^*2*^ = 45%), scores 18–30 (OR: 1.12, 95% CI: 1.00–1.26, *P* = 0.060, *I*^*2*^ = 0%) and scores >30 (OR: 0.69, 95% CI: 0.45–1.07, *P* = 0.100, *I*^*2*^ = 81%) as MBC patients (Fig. 2A, B and 2C).

## 4 Discussion

This is the first systematic review and meta-analysis assessing the reliability of using the 21-gene expression assay for MBC diagnosed with ER-positive, HER2-negative early breast carcinoma. At present, few studies have outlined the clinical utility of using genomic signatures to guide therapeutic decision-making in the setting of MBC patients. The results from this analysis suggest there are similar results anticipated in breast cancer patients irrespective of gender, despite the genetic signature being only previously validated in female breast cancer patients. Consequently, in the setting of diagnosing an early-stage, ER-positive, HER2-negative carcinoma in a male patient, this study provides provisional data supporting the use of the 21-gene expression assay to provide prognostication and predict benefit of prescribing chemoendocrine agents. However, caution is required when interpreting such results: Overall, MBC was associated with more advanced tumour staging and grade, and perhaps failure to match cases provides explanation for the increased propensity for MBC to develop RS > 30 relative to female patients with the ER-positive and HER2-negative disease. However, the biological differences between female and male patient with ER-positive, HER2-negative disease is challenging to extrapolate from this data, meaning further validation of the 21-gene expression assay is required before implementation as routine into multidisciplinary discussion around MBC management. Moreover, we note that other multigene risk substratification panels, such as the 70-gene expression assay, have been assessed for application to MBC and may be as relevant for providing prognostication for male patients [19].

Overall, MBC were as likely as female patients to have 21-gene assay scores <18 (OR: 1.04, 95% CI: 0.94–1.16) and scores 18–30 (OR: 1.12, 95% CI: 1.00–1.26). This may be perceived to be somewhat surprising: The algorithm for 21-gene assay testing is derived from an equation which incorporates the expression of genes representing ER, PgR, and HER2/neu as continuous parameters through reverse-transcription quantitative polymerase chain reaction products [10], where subsequent determined values for steroid hormones (i.e.: ER and PgR) are negatively deducted from the total calculated in the algorithm. Indication for 21-gene expression assay testing includes ER-status as a dichotomous parameter, and it is well described that MBC are more likely to develop ER-positive breast cancers than their female counterparts: In their analysis of available ER data from the Surveillance, Epidemiology and End Results database, Anderson et al. established that 92.4% of MBC are ER-positive (2575/2788) compared to 77.5% of female patients (344,406/444,558) [25]. Similarly, data from Cardoso et al. highlighted that 99.3% of MBC are ER-positive [26]. Nevertheless, we must appreciate that all patients undergoing 21-gene expression assay testing must have been originally classed as ER-positive, despite ER-expression (and positivity) occurring along a spectrum [27]. Interestingly, Muftah et al. previously described a bimodal distribution of ER-positivity in their large analysis of 3649 female patients with breast cancer [28]. The authors reported 92.2% of all included patients had either strongly positive (≥70%) or negative (<1%) ER expression, highlighting the bimodal distribution of this steroid hormone at a cellular level. Therefore, when considering the bimodal expression of ER in female breast cancers, it is reasonable that all female patients indicated to undergo 21-gene expression assay testing are strongly ER-positive, which explains the comparable results for female and MBC patients undergoing 21-gene expression assay testing.

In this study, the data included in this analysis may be subject to several ascertainment and selection biases, which impact the results observed: MBC patients selected to undergo 21-gene expression assay testing are likely to be cases with borderline aggressive clinicopathological features, where guidance surrounding therapeutic decision making is required before committing the patient to adjuvant chemotherapy. Therefore, as previously outlined, failure to provide stage-matched comparisons is likely to account for these findings and adds uncertainty surrounding conclusions drawn from this data supporting the indication for early-stage MBC patients to undergo genomic substratification using multigene panels. Thus, the authors highlight the importance of the data from the current study to highlight the need for further studies evaluating the suitability of the 21-gene assay for implementation into the management paradigm for MBC in clinical practice.

These results indicate that MBC patients are equally as likely to have scores >30 (OR: 0.69, 95% CI: 0.45–1.07) than their female counterparts. Once again, this is an interesting finding: As previously outlined, MBC tend to be molecularly identified as Luminal A intrinsic subtype (ER-positive, PgR-positive, HER2-negative, low-grade). Using the PAM-50 gene signature, a second-generation multigene expression assay, greater than 90% of MBC have molecular profiles consistent with Luminal cancers [29]. The work of Sanchez-Munoz et al. indicates that MBC are more accustom to fitting with the intrinsic biological profile of Luminal B disease (60% Luminal B: 40/67, 30% Luminal A: 20/67, 10% HER2-enriched: 7/67, 0% basal-like: 0/67). This does differ substantially from the allocation of intrinsic biological subtypes in female patients, as outlined by Parker et al. [30], in their seminal study outlining the value of PAM-50 gene in predicting risk-recurrence (35% Luminal B: 269/761, 22% Luminal A: 168/761, 16% HER2-enriched: 120/761, 17% basal-like: 128/761, 10% normal-like: 76/761). However, when addressing the proportions of Luminal B-like cancers in those with ER-positive, HER2-negative cancers, the proportion is similar for both groups (MBC: 67%, 40/60, female cancers: 62%, 269/437), providing rationale for the comparable 21-gene expression scores observed in both genders in this study. With this in mind, it is possible that MBC may be as likely as females to have 21-gene expression scores greater than 30, however the previously described ascertainment biases impact interpretation of these results. Once again, attention must be brought to the failure to match female and MBC in this analysis, which limits the reliability of results regarding the 21-gene expression assay in predicting scores >30.

While the 21-gene expression assay provides sensitive recurrence risk profile for female patients, caution must be taken when interpreting results for MBC patients. Within the 21-gene assay, the expression profiles of ER, HER2, proliferation, and invasion oncogenes are combined to calculate the composite ‘recurrence score’ While these carefully selected target genes are considered most appropriate for female patients, the important role of the androgen-receptor (AR) in MBC falls considerably short of being in consideration for inclusion in the signature. Like the ER, AR is a member of the steroid-hormone receptor superfamily and can be expressed in high concentrations on breast cancer tissue [31,32]. Overall, 90% of MBC patients express AR-receptors on the surface of their tumour cells [29,33], which may serve as potential therapeutic targets [34], as is routinely observed in the treatment of prostatic adenocarcinoma [35]. ER-positive breast cancers are significantly more likely to be AR-positive than ER-negative tumours [36], with some studies suggesting AR status is a correlate of ER-alpha/PgR signaling [37]. However, AR expression in ER-positive breast cancer has been observed to antagonize ER-alpha in pre-clinical studies, while agonizing and upregulating the ER-beta signaling pathway [38]. The presence of ER-beta signaling has been illustrated to inhibit the translational activity of ER-alpha, indicating that the presence of AR indirectly influences the activity of the ER-alpha signaling pathway. Moreover, recent data suggests that high AR expression may be correlated with tamoxifen resistance [39], suggesting that AR-expression profiles may be crucial in MBC patients being treated with first-line anti-endocrine agents. However, this conundrum is not reflected clinically, with a meta-analysis from Vera-Badillo et al. reporting enhanced clinical outcomes for ER-positive/AR-positive cancers versus their counterparts [36]. Thus, further clinical interrogation of the role of the AR within the clinical context of ER-positive/HER2-negative invasive MBC is warranted before considering making personalised adaptations to conventional genomic signatures to encompass MBC patients.

This meta-analysis is subject to several limitations. To reiterate, the results of this meta-analysis should be interpreted with caution as patients diagnosed with MBC who were included in these analyses had increased tumour burden in both the breast and axilla, as well as higher tumour grade (all *P* < 0.001). In the absence of stage matching for male and female breast cancer, it proves difficult to provide relevant conclusion with respect to 21-gene assay scores. Potential ascertainment biases surrounding the selection of MBC patients undergoing 21-gene testing may also limit these results. Of note, each of the included studies are retrospective in design, which inevitably renders them subject to the inherent limitations of ascertainment, confounding, and selection biases. On account of the design and nature of this synthetic review, it is not feasible to control for these limiting factors. Furthermore, over 98% of patients included in the current analysis were taken from the Surveillance, Epidemiology and End Results (SEER) and National Cancer (NCDB) databases, limiting the validity of conclusions drawn from this analysis [23,24]. In spite of these limitations, this analysis is the first to integrate real world data assessing the clinical utility of the 21-gene expression assay in MBC patients and adds to the current vogue hoping to expand indications for the genomic assay in clinical practice [[40], [41], [42], [43]].

In conclusion, the data from this systematic review and meta-analysis suggests there is similar anticipated scores for both male and female breast cancer patients undergoing the 21-gene expression assay for ER-positive, HER2-negative breast cancer. However, the authors wish to highlight that the results of the 21-gene expression assay in male patients should be interpreted with caution due to the failure of this analysis to appropriately stage match these patients and the potential ascertainment biases surrounding MBC patient selection for testing. Future studies validating the role of the 21-gene expression assay may consider the validation of this genomic assay in a MBC population in order to aid consensus in relation to the clinical utility of this biomarker in clinical practice.

## Sources of funding

MGD, CMM, LB and EK received funding from the National Breast Cancer Research Institute, Ireland.
